# The Impact of an Exceptional Lung Allocation Score on Organ Access of Failing Pulmonary Arterial Hypertension Patients – A Eurotransplant Experience

**DOI:** 10.3389/ti.2025.15013

**Published:** 2025-10-28

**Authors:** S. Schwarz, S. Vogelaar, C. Knoop, F. Dzubur, G. Warnecke, L. Bogyo, T. Stupnik, L. Seghers, P. Evrard, R. Schramm, B. Gieszer, J. Gummert, M. Harlander, A. Benazzo, P. Jaksch, K. Hoetzenecker

**Affiliations:** ^1^ Department of Thoracic Surgery, Medical University of Vienna, Vienna, Austria; ^2^ Eurotransplant International Foundation, Leiden, Netherlands; ^3^ Department of Chest Medicine, Hôpital Erasme, Brussels, Belgium; ^4^ Clinical Center for Pulmonary Diseases, University Hospital Centre Zagreb, Zagreb, Croatia; ^5^ Department of Cardiac Surgery, University Hospital of Schleswig-Holstein, Kiel, Germany; ^6^ Department of Thoracic Surgery, National Institute of Oncology, Semmelweis University, Budapest, Hungary; ^7^ Department of Thoracic Surgery, University Medical Center, Ljubljana, Slovenia; ^8^ Department of Pulmonary Medicine, Erasmus University Medical Center, Rotterdam, Netherlands; ^9^ Department of Intensive Care, Centre Hospitalier Universitaire Université Catholique de Louvain, Namur Godinne, Belgium; ^10^ Department of Thoracic and Cardiovascular Surgery, Heart and Diabetes Center North Rhine-Westphalia, Ruhr-University Bochum, Bad Oeyenhausen, Germany; ^11^ Department of Pulmonary Diseases, University Medical Center, Ljubljana, Slovenia; ^12^ Department of Thoracic Surgery, Vanderbilt University Medical Center, Nashville, TN, United States

**Keywords:** lung allocation score, pulmonary arterial hypertension (PAH), lung transplant, organ allocation, waiting list

Dear Editors,

PAH patients remain a challenging diagnosis group in lung transplantation. Cardiac decompensation can render patients non-transplantable resulting in higher rates of waitlist deaths. Therefore, timely transplantation is of utmost importance in this patient group. However, capturing the true urgency of PAH patients is challenging in a system primarily designed for parenchymal pulmonary diseases. Patients suffering from pulmonary-vascular diseases usually have low LAS scores due to preserved pulmonary function parameters and gas exchange. The eLAS system was introduced to account for this discrepancy in the ET region. A board of independent judges, reviews clinical parameters as well as trajectories of disease severity for each eLAS application on an individual basis. For PAH patients, eLAS can be granted for patients with cardiac index <2 L/m^2^ and right atrial pressure >15 mmHg, a bilirubin increase by 50%/abnormal, a creatinine increase by >50%/abnormal and for PAH patients on awake ECMO. If an eLAS application is accepted by the LAS review board, the patient’s conventional LAS is replaced by a score corresponding to the 95-99th percentile of all patients listed in the Eurotransplant region. To assess how this system impacts waitlist mortality and early survival in PAH patients, we analyzed a total of 241 PAH patients receiving double lung transplant using ET data (Figure 1). Patients in the eLAS group tended to be younger (median 40y vs. 46y; p = 0.017) and more often female compared to the LAS group (72.1% vs. 59.3%; p = 0.076). Systolic PAP (100 vs. 84.5 mmHg; p = 0.022), mean PAP (65 vs. 54 mmHg; p = 0.011) and central venous pressure (12.5 vs. 10 mmHg; p = 0.017) were significantly higher in the eLAS group. Post-transplant mortality was highest in patients transplanted after declined eLAS (90-day: 36.8%) compared to 19.2% in patients with a valid eLAS and 10.1% in the LAS group (p = 0.018). Differences in 1-year-mortality did not reach statistical significance.

**FIGURE 1:**
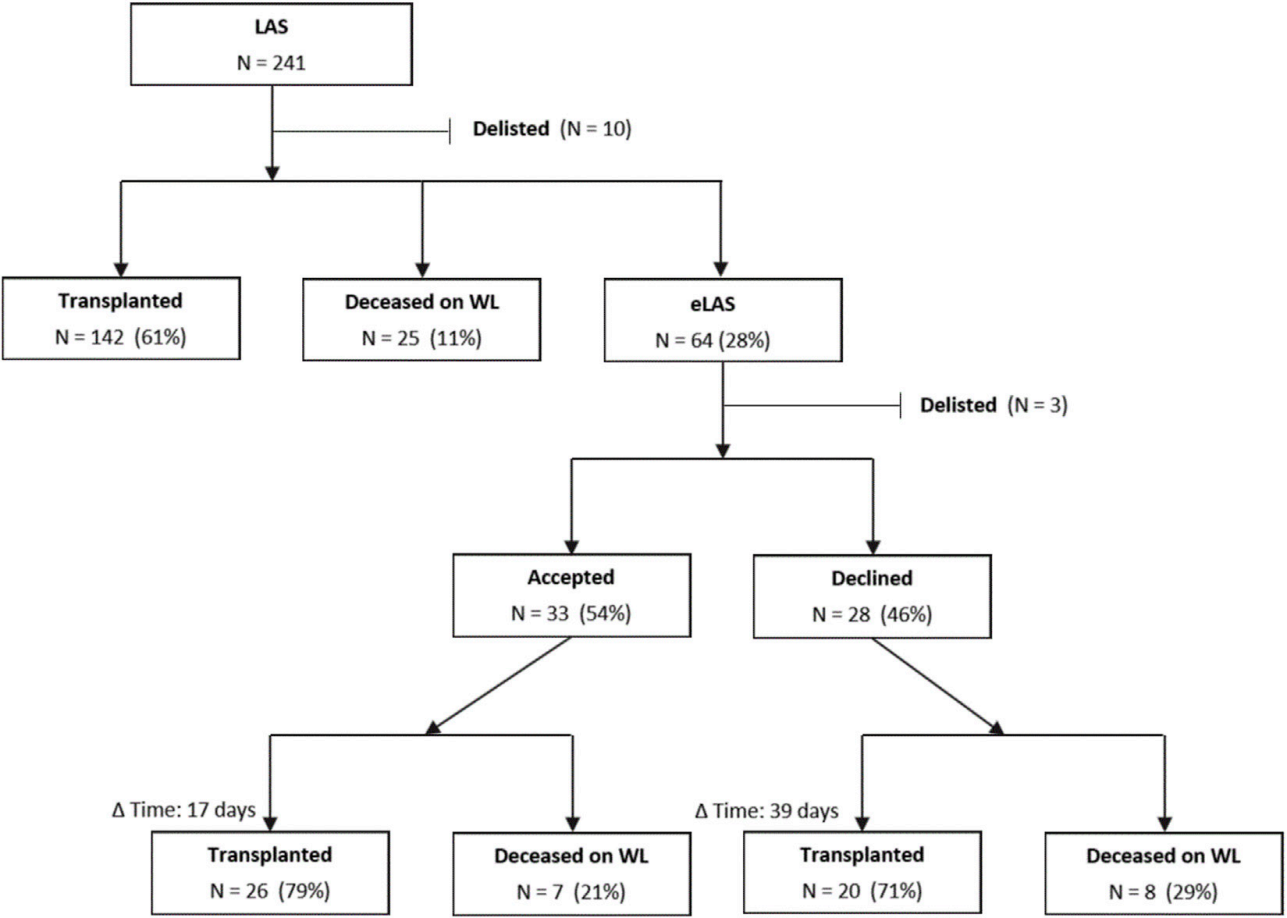
Flowchart of PAH patients through the LAS/eLAS system. (Abbreviations: d, day; Δ Time, Days between listing and event, i.e., either transplantation or death; eLAS, Exceptional lung allocation score; LAS, Lung allocation score; WL, Waitlist; y, Year).

The implementation of the ET LAS system in 2011 is generally considered a success. It facilitated cross-border sharing of donor lungs for high-urgent recipients, thus reducing waitlist mortality and median waiting time [1]. Especially patients with restrictive lung diseases profited from a LAS-based allocation. In contrast, PAH patients were the only group showing worse survival after LAS implementation in Germany [1]. In addition, PAH patients have the highest waitlist mortality and second lowest chance of being transplanted within 1 year in the ET region.

One of the important differences between failing PAH patients and patients suffering from parenchymal lung disease is the complexity to bridge patients for a prolonged time. In the majority of cases with non-PAH lung disease, VV-ECMO is sufficient, whereas PAH patients require a VA mode for right ventricle unloading and cardiac support. VV-ECMO is far less invasive and can be performed for extended periods of time, while VA-ECMO is known to entail higher risks for bleeding or thromboembolic complications. Indeed, PAH patients have the lowest transplantation rate of ECLS-bridged patients. Therefore, an ideal organ allocation system should aim to avoid the need for ECLS bridging by assigning higher urgency to PAH patients while they are still stable. It seems that the current eLAS system is only partially successful in this regard, as eLAS can only be requested for PAH patients already in right heart failure.

Recently, important advances have been made in assessing decompensation risk of PAH patients. Parameters suggested by the latest ERS guidelines include WHO-FC, 6MWD, biomarkers and cardiac imaging (e.g., changes of RV dimension, RV fractional area change, RV free wall strain, tricuspid annular plane systolic excursion, tricuspid regurgitation, TAPSE/sPAP, RA area) [2]. With increasing evidence for these factors in the general PAH population, they might also be useful to incorporate in lung allocation scores. This is further underlined by a study by Vicaire et al., showing that high-risk PAH patients according to COMPERA 2.0, REVEAL Lite 2 as well as ESC/ERS guidelines at the time of listing had poor outcomes after LTx, emphasizing the need for early referral to a lung transplant center [3].

Currently, an accepted eLAS request results in scores between the 95th and 99th percentile. This resulted in a timely transplantation of 79% of patients in our analysis. This is unique and underlines the importance of being able to apply for an exceptional score, as the majority of these patients would have died. Of note, in 26/33 patients in very critical situations (based on the eLAS requirements) were rescued with an acceptable 1-year survival. In contrast, almost half of eLAS requests in our cohort were rejected by the LAS-board as they did not meet preset criteria, leaving these patients on the list with a regular LAS. Interestingly, many of them were transplanted, but with almost quadrupled 90-day mortality. This observation aligns with Wille et al. who found significantly lower 1-year survival for group B patients with denied exceptional LAS requests in the US. [4].

As LAS calculation aims to balance pre-transplant likelihood of death and post-transplant survival, some have argued that therapeutic benefit is not favourable in many PAH patients, not meriting allocation of scarce donor organs. However, PAH patients are currently at an unfair disadvantage on both sides of this equation. It has been shown that the LAS calculation actually underestimates pre-transplant mortality of PAH patients awaiting transplantation [5]. This is especially relevant since risk of waitlist mortality factors into the ET-LAS calculation with twice the weight of post-transplant likelihood of survival. At the same time, post-transplant survival has significantly improved in recent years and is comparable to other underlying diseases in high-volume centers, especially by the use of postoperative ECMO prolongation strategies. Survival conditional on survival to 3 months is well-known to exceed all other diagnosis groups except CF [6]. Taken together, PAH patients make excellent transplant candidates who deserve equity in organ allocation.

Our investigation has several limitations. Being a retrospective analysis based on registry data, it can contain miscoded data or missing values. We lacked clinical information on PAH patients at the time of listing and their trajectories until they were transplanted or died on the waitlist. In addition, it is unknown how often LAS scores have been updated during the waiting time. Detailed measurements of patient hemodynamic impairment are not captured by ET, preventing competing risk analysis of known parameters predicting cardiac failure in PAH**.** Also, specific reasons for the refusal of an eLAS request by the board were not available. As our cohort includes patients transplanted over the course of almost 8 years, the analysis could also be prone to era effects.

In conclusion, allocation requires a balanced reflection of urgency. Our analysis suggests that the current ET allocation system is not optimal for PAH patients, who are underprivileged compared to other indication groups. The option to grant an eLAS for PAH patients addresses some of the flaws but is not specific enough to reduce waitlist mortality in this highly specific subgroup of patients. Further refinement of the ET-LAS, additional business rules or a revision of the eLAS system are warranted to ensure equal access to life-saving transplantation for PAH patients.

## Data Availability

Data not available due to ethical/legal restrictions.

## Abbreviations

ET, Eurotransplant; PAH, Pulmonary arterial hypertension; LAS, Lung allocation score; eLAS, Exceptional lung allocation score; PAP, Pulmonary artery pressure; IQR, Inter-quartile range.
